# Supplementary figures and images for: Genome-Engineered mpkCCDc14 Cells as a New Resource for Studying AQP2
Source: Int J Mol Sci. 2023 Jan 14;24(2):1684. doi: 10.3390/ijms24021684 (PMC9866188; doi:10.3390/ijms24021684)

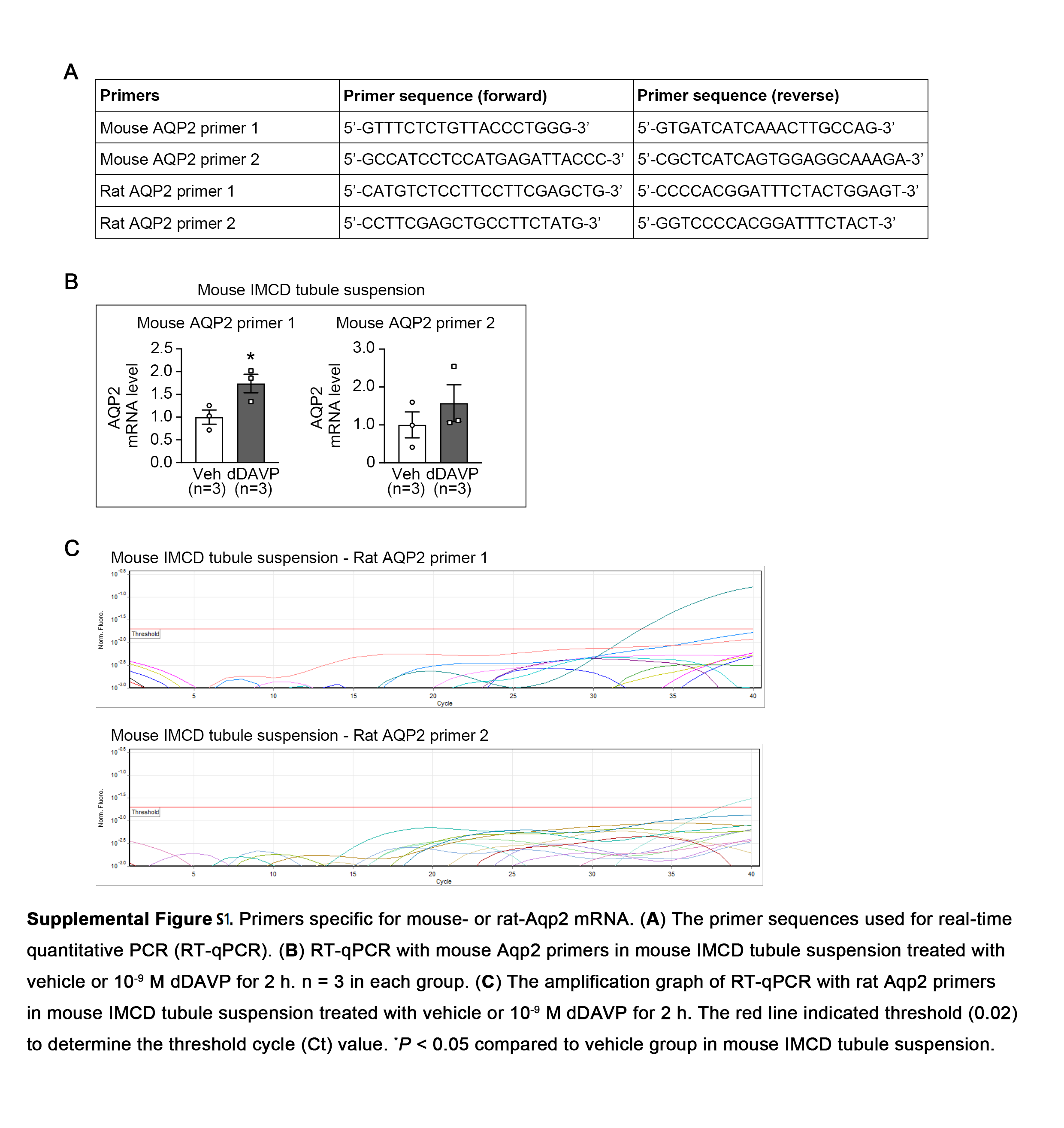

Supplement: Supplementary file 1 [file ijms-24-01684-s001.zip › Supplementary Figure S1.tif]

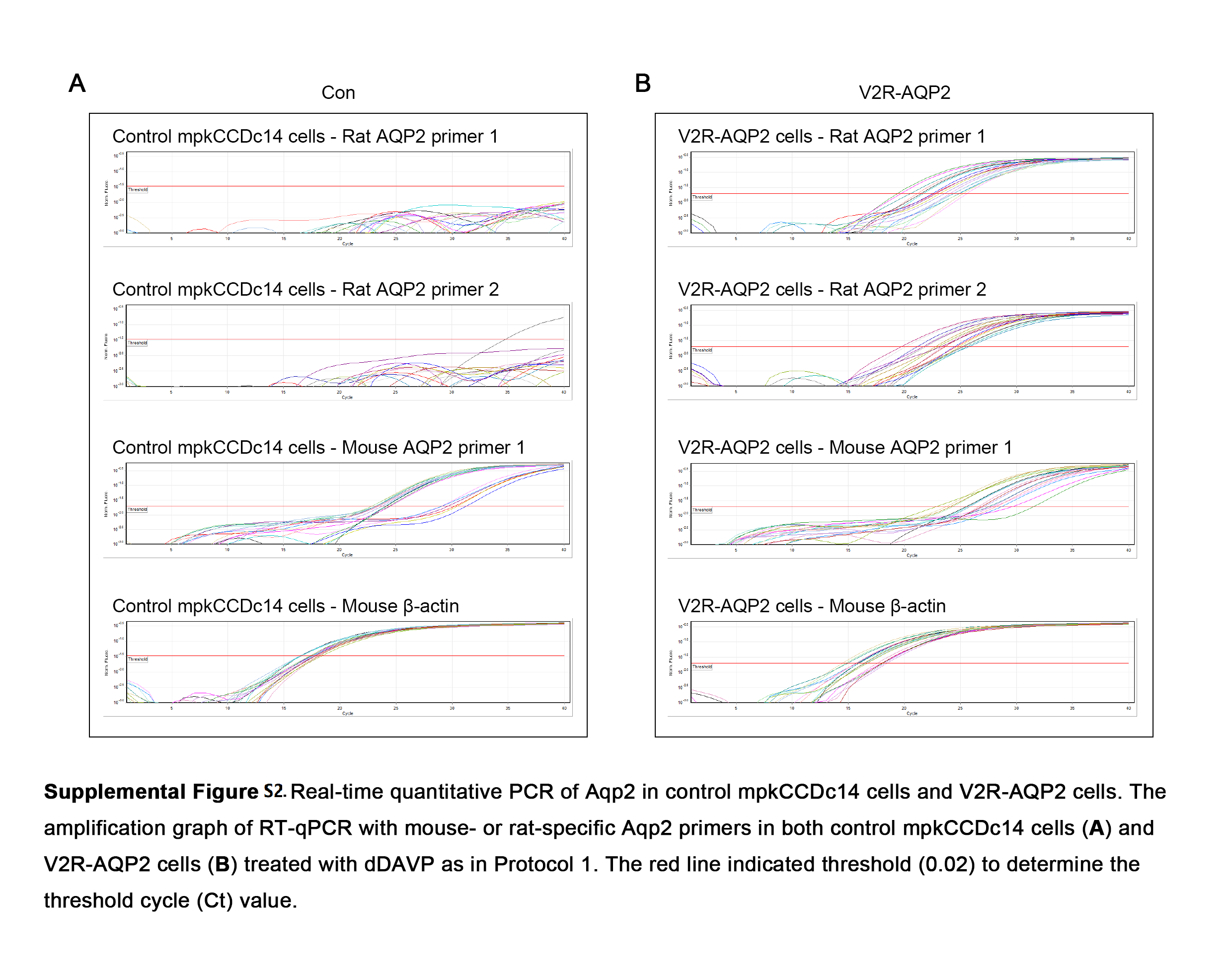

Supplement: Supplementary file 1 [file ijms-24-01684-s001.zip › Supplementary Figure S2.tif]

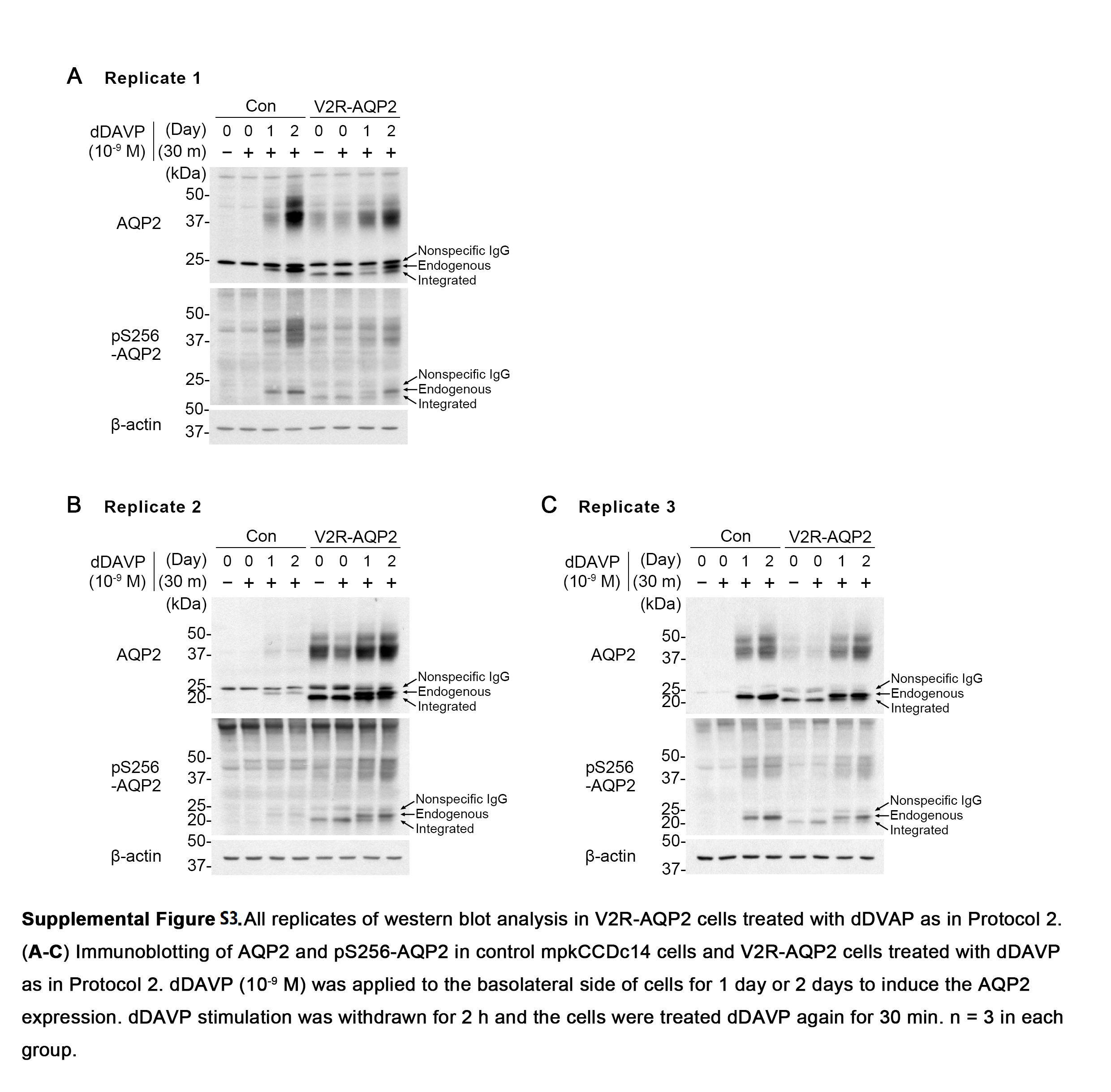

Supplement: Supplementary file 1 [file ijms-24-01684-s001.zip › Supplementary Figure S3.tif]
